# Supplementary material for: Elevated Interleukin-6 Levels in the Circulation and Peritoneal Fluid of Patients with Ovarian Cancer as a Potential Diagnostic Biomarker: A Systematic Review and Meta-Analysis
Source: J Pers Med. 2021 Dec 9;11(12):1335. doi: 10.3390/jpm11121335 (PMC8704427; doi:10.3390/jpm11121335)
Supplement: Supplementary file 1 [file jpm-11-01335-s001.zip › jpm-1446040-supplementary/Supplementary Table S2 - STROBE check for the selected studies.pdf]

Supplementary Table S2. Selected studies with STROBE eligibility criteria

No.	Author	Year	Country	Participant selection	Introduction	Study design	Sample handling protocols	Results	Outcome
1.	Sanna et al.(1)	2021	Italy	✓	✓	✓	✓	✓	✓
2.	Micheli et al.(2)	2020	Brazil	✓	✓	✓	✓	✓	✓
3.	de Lima et al.(3)	2020	Brazil	✓	✓	✓	✓	✓	✓
4.	Rodrigues et al.(4)	2020	Brazil	✓	✓	✓	✓	✓	✓
5.	Kampan et al. (5)	2020	Australia	✓	✓	✓	✓	✓	✓
6.	Wertel et al.(6)	2020	Poland	✓	✓	✓	✓	✓	✓
7.	Zhang et al.(7)	2020	China	✓	✓	✓	✓	✓	✓
8.	Crispim et al.(8)	2020	Brazil	✓	✓	✓	✓	✓	✓
9.	Shi et al(9)	2019	China	✓	✓	✓	✓	✓	✓
10.	Li et al.(10)	2019	China	✓	✓	✓	✓	✓	✓
11.	Dalal et al.(11)	2018	India	✓	✓	✓	✓	✓	✓
12.	Han et al.(12)	2018	USA	✓	✓	✓	✓	✓	✓
13.	Kumar et al.(13)	2017	Italy	✓	✓	✓	✓	✓	✓
14.	Martins-Filho et al.(14)	2017	Brazil	✓	✓	✓	✓	✓	✓

15.	Cantón-Romeo et al.(15)	2017	Mexico	✓	✓	✓	✓	✓	✓
16.	Sanguinette et al. (16)	2017	Brazil	✓	✓	✓	✓	✓	✓
17.	Hao et al.(17)	2016	China	✓	✓	✓	✓	✓	✓
18.	Mikuła-Pietrasik et al.(18)	2016	USA	✓	✓	✓	✓	✓	✓
19.	Chudecka-Głaz et al. (19)	2015	Poland	✓	✓	✓	✓	✓	✓
20.	Block et al.(20)	2015	USA	✓	✓	✓	✓	✓	✓
21.	Matsuo et al.(21)	2015	USA	✓	✓	✓	✓	✓	✓
22.	Ose et al.(22)	2015	Europe	✓	✓	✓	✓	✓	✓
23.	Lane et al.(23)	2015	Canada	✓	✓	✓	✓	✓	✓
24.	Trabert et al.(24)	2014	USA	✓	✓	✓	✓	✓	✓
25.	Cândido et al.(25)	2013	Brazil	✓	✓	✓	✓	✓	✓
26.	Poole et al.(26)	2013	USA	✓	✓	✓	✓	✓	✓
27.	Dobrzycka et al.(27)	2013	Poland	✓	✓	✓	✓	✓	✓
28.	Matte et al. (28)	2012	Canada	✓	✓	✓	✓	✓	✓
29.	Autelitano et al.(29)	2012	Australia	✓	✓	✓	✓	✓	✓
30.	Clendenen et al.(30)	2011	USA	✓	✓	✓	✓	✓	✓

31.	Sen et al.(31)	2011	Turkey	✓	✓	✓	✓	✓	✓
32.	Yigit et al(32)	2011	Netherland	✓	✓	✓	✓	✓	✓
33.	Edgell et al.(33)	2010	Australia	✓	✓	✓	✓	✓	✓
34.	Nowak et al.(34)	2010	Poland	✓	✓	✓	✓	✓	✓
35.	Napoletano et al.(35)	2010	Italy	✓	✓	✓	✓	✓	✓
36.	Tsai-Turton et al.(36)	2009	USA	✓	✓	✓	✓	✓	✓
37.	Amonkar et al.(37)	2009	USA	✓	✓	✓	✓	✓	✓
38.	Macciò et al.(38)	2009	Italy	✓	✓	✓	✓	✓	✓
39.	Lutgendorf et al.(39)	2008	France	✓	✓	✓	✓	✓	✓
40.	Kavask et al.(40)	2008	Canada	✓	✓	✓	✓	✓	✓
41.	Lambeck et al.(41)	2007	USA	✓	✓	✓	✓	✓	✓
42.	Chechlinska et al.(42)	2007	Poland	✓	✓	✓	✓	✓	✓
43.	Costanzo et al.(43)	2005	USA	✓	✓	✓	✓	✓	✓
44.	Macciò et al.(44)	2005	Italy	✓	✓	✓	✓	✓	✓
45.	Gorelik et al.(45)	2005	Italy	✓	✓	✓	✓	✓	✓
46.	Daraï et al.(46)	2003	France	✓	✓	✓	✓	✓	✓
47.	Dobryszyca et al.(47)	1999	Poland	✓	✓	✓	✓	✓	✓

48.	Tempfer et al. (48)	1996	Austria	✓	✓	✓	✓	✓	✓
49.	Scambia et al.(49)	1995	Italy	✓	✓	✓	✓	✓	✓
50.	Plante et al.(50)	1994	Canada	✓	✓	✓	✓	✓	✓
51.	Schröder et al(51)	1994	Germany	✓	✓	✓	✓	✓	✓
52.	van der Zee et al.(52)	1994	Belgium	✓	✓	✓	✓	✓	✓
53.	Moradi et al.(53)	1993	USA	✓	✓	✓	✓	✓	✓
54.	Gastl et al.(54)	1993	USA	✓	✓	✓	✓	✓	✓
55.	Berek et al(55)	1991	USA	✓	✓	✓	✓	✓	✓

1. Sanna E, Tanca L, Cherchi C, Gramignano G, Oppi S, Chiai MG, et al. Decrease in Neutrophil-to-Lymphocyte Ratio during Neoadjuvant Chemotherapy as a Predictive and Prognostic Marker in Advanced Ovarian Cancer. Diagnostics (Basel). 2021;11(7).
2. Micheli DC, Jammal MP, Martins-Filho A, Côrtes JRXDM, Souza CND, Nomelini RS, et al. Serum cytokines and CXCR2: potential tumour markers in ovarian neoplasms. Biomarkers. 2020;25(6):474-82.
3. de Lima CA, Silva Rodrigues IS, Martins-Filho A, Côbo Micheli D, Martins Tavares-Murta B, Candido Murta EF, et al. Cytokines in peritoneal fluid of ovarian neoplasms. J Obstet Gynaecol. 2020;40(3):401-5.
4. Rodrigues ISS, Martins-Filho A, Micheli DC, Lima CA, Tavares-Murta BM, Murta EFC, et al. IL-6 and IL-8 as Prognostic Factors in Peritoneal Fluid of Ovarian Cancer. Immunol Invest. 2020;49(5):510-21.
5. Kampan NC, Madondo MT, Reynolds J, Hallo J, McNally OM, Jobling TW, et al. Pre-operative sera interleukin-6 in the diagnosis of high-grade serous ovarian cancer. Sci Rep. 2020;10(1):2213.
6. Wertel I, Suszczyk D, Pawłowska A, Bilska M, Chudzik A, Skiba W, et al. Prognostic and Clinical Value of Interleukin 6 and CD45(+)CD14(+) Inflammatory Cells with PD-L1(+)/PD-L2(+) Expression in Patients with Different Manifestation of Ovarian Cancer. J Immunol Res. 2020;2020:1715064.
7. Zhang H, Chen C, Wang S, Li X, Fan T. Efficacy of bevacizumab combined with nedaplatin in the treatment of ovarian cancer and its effects on tumor markers and immunity of patients. J buon. 2020;25(1):80-6.
8. Crispim PCA, Jammal MP, Antão PKA, Micheli DC, Tavares-Murta BM, Murta EFC, et al. IL6, IL8, and IL10 in the distinction of malignant ovarian neoplasms and endometriomas. Am J Reprod Immunol. 2020;84(6):e13309.

9. Shi J, Huo R, Li N, Li H, Zhai T, Li H, et al. CYR61, a potential biomarker of tumor inflammatory response in epithelial ovarian cancer microenvironment of tumor progress. BMC Cancer. 2019;19(1).
10. Li G, Zhang K, Gong F, Jin H. A study on changes and clinical significance of blood glucose, blood lipid and inflammation in patients with ovarian cancer. J buon. 2019;24(6):2322-6.
11. Dalal V, Kumar R, Kumar S, Sharma A, Kumar L, Sharma JB, et al. Biomarker potential of IL-6 and VEGF-A in ascitic fluid of epithelial ovarian cancer patients. Clin Chim Acta. 2018;482:27-32.
12. Han C, Bellone S, Siegel ER, Altwerger G, Menderes G, Bonazzoli E, et al. A novel multiple biomarker panel for the early detection of high-grade serous ovarian carcinoma. Gynecologic Oncology. 2018;149(3):585-91.
13. Inflammatory and Nutritional Serum Markers as Predictors of Peri-operative Morbidity and Survival in Ovarian Cancer. Anticancer Research. 2017;37(7).
14. Martins-Filho A, Jammal MP, Micheli DC, Tavares-Murta BM, Etchebehere RM, Murta EFC, et al. Role of Intracystic Cytokines and Nitric Oxide in Ovarian Neoplasms. Scandinavian Journal of Immunology. 2017;86(6):462-70.
15. Cantón-Romero JC, Miranda-Díaz AG, Bañuelos-Ramírez JL, Carrillo-Ibarra S, Sifuentes-Franco S, Castellanos-González JA, et al. Markers of Oxidative Stress and Inflammation in Ascites and Plasma in Patients with Platinum-Sensitive, Platinum-Resistant, and Platinum-Refractory Epithelial Ovarian Cancer. Oxid Med Cell Longev. 2017;2017:2873030.
16. Sanguinete MMM, Oliveira PH, Martins-Filho A, Micheli DC, Tavares-Murta BM, Murta EFC, et al. Serum IL-6 and IL-8 Correlate with Prognostic Factors in Ovarian Cancer. Immunol Invest. 2017;46(7):677-88.
17. Hao CJ, Li J, Liu P, Li XL, Hu YQ, Sun JC, et al. Effects of the balance between type 1 and type 2 T helper cells on ovarian cancer. Genet Mol Res. 2016;15(2).
18. Mikuła-Pietrasik J, Uruski P, Szubert S, Moszyński R, Szpurek D, Sajdak S, et al. Biochemical composition of malignant ascites determines high aggressiveness of undifferentiated ovarian tumors. Medical Oncology. 2016;33(8):94.
19. Chudecka-Głaz AM, Cymbaluk-Płoska AA, Menkiszak JL, Pius-Sadowska E, Machaliński BB, Sompolska-Rzechuła A, et al. Assessment of selected cytokines, proteins, and growth factors in the peritoneal fluid of patients with ovarian cancer and benign gynecological conditions. Onco Targets Ther. 2015;8:471-85.
20. Block MS, Maurer MJ, Goergen K, Kalli KR, Erskine CL, Behrens MD, et al. Plasma immune analytes in patients with epithelial ovarian cancer. Cytokine. 2015;73(1):108-13.
21. Matsuo K, Hasegawa K, Yoshino K, Murakami R, Hisamatsu T, Stone RL, et al. Venous thromboembolism, interleukin-6 and survival outcomes in patients with advanced ovarian clear cell carcinoma. Eur J Cancer. 2015;51(14):1978-88.
22. Ose J, Schock H, Tjønneland A, Hansen L, Overvad K, Dossus L, et al. Inflammatory Markers and Risk of Epithelial Ovarian Cancer by Tumor Subtypes: The EPIC Cohort. Cancer Epidemiol Biomarkers Prev. 2015;24(6):951-61.
23. Lane D, Matte I, Garde-Granger P, Laplante C, Carignan A, Rancourt C, et al. Inflammation-regulating factors in ascites as predictive biomarkers of drug resistance and progression-free survival in serous epithelial ovarian cancers. BMC Cancer. 2015;15(1).

24. Trabert B, Pinto L, Hartge P, Kemp T, Black A, Sherman ME, et al. Pre-diagnostic serum levels of inflammation markers and risk of ovarian cancer in the Prostate, Lung, Colorectal and Ovarian Cancer (PLCO) Screening Trial. Gynecologic Oncology. 2014;135(2):297-304.
25. Cândido EB, Silva LM, Carvalho AT, Lamaita RM, Filho RM, Cota BD, et al. Immune response evaluation through determination of type 1, type 2, and type 17 patterns in patients with epithelial ovarian cancer. Reprod Sci. 2013;20(7):828-37.
26. Poole EM, Lee IM, Ridker PM, Buring JE, Hankinson SE, Tworoger SS. A Prospective Study of Circulating C-Reactive Protein, Interleukin-6, and Tumor Necrosis Factor α Receptor 2 Levels and Risk of Ovarian Cancer. American Journal of Epidemiology. 2013;178(8):1256-64.
27. Dobrzycka B, Mackowiak-Matejczyk B, Terlikowska KM, Kulesza-Bronczyk B, Kinalski M, Terlikowski SJ. Serum levels of IL-6, IL-8 and CRP as prognostic factors in epithelial ovarian cancer. Eur Cytokine Netw. 2013;24(3):106-13.
28. Matte I, Lane D, Laplante C, Rancourt C, Piché A. Profiling of cytokines in human epithelial ovarian cancer ascites. Am J Cancer Res. 2012;2(5):566-80.
29. Autelitano DJ, Raineri L, Knight K, Bannister K, Rice GE. Performance of a multianalyte test as an aid for the diagnosis of ovarian cancer in symptomatic women. Journal of Translational Medicine. 2012;10(1):45.
30. Clendenen TV, Lundin E, Zeleniuch-Jacquotte A, Koenig KL, Berrino F, Lukanova A, et al. Circulating Inflammation Markers and Risk of Epithelial Ovarian Cancer. Cancer Epidemiology Biomarkers & Prevention. 2011;20(5):799-810.
31. Sen S, Kuru O, Akbayir O, Oguz H, Yasasever V, Berkman S. Determination of serum CRP, VEGF, Leptin, CK-MB, CA-15-3 and IL-6 levels for malignancy prediction in adnexal masses. Journal of the Turkish German Gynecological Association. 2011;12(4):214-9.
32. Yigit R, Figdor CG, Zusterzeel PL, Pots JM, Torensma R, Massuger LF. Cytokine analysis as a tool to understand tumour-host interaction in ovarian cancer. Eur J Cancer. 2011;47(12):1883-9.
33. Edgell T, Martin-Roussety G, Barker G, Autelitano DJ, Allen D, Grant P, et al. Phase II biomarker trial of a multimarker diagnostic for ovarian cancer. J Cancer Res Clin Oncol. 2010;136(7):1079-88.
34. Nowak M, Glowacka E, Szpakowski M, Szyllo K, Malinowski A, Kulig A, et al. Proinflammatory and immunosuppressive serum, ascites and cyst fluid cytokines in patients with early and advanced ovarian cancer and benign ovarian tumors. Neuro Endocrinol Lett. 2010;31(3):375-83.
35. Napoletano C, Bellati F, Landi R, Pauselli S, Marchetti C, Visconti V, et al. Ovarian cancer cytoreduction induces changes in T cell population subsets reducing immunosuppression. J Cell Mol Med. 2010;14(12):2748-59.
36. Tsai-Turton M, Santillan A, Lu D, Bristow RE, Chan KC, Shih I-M, et al. p53 autoantibodies, cytokine levels and ovarian carcinogenesis. Gynecologic Oncology. 2009;114(1):12-7.
37. Amonkar SD, Bertenshaw GP, Chen T-H, Bergstrom KJ, Zhao J, Seshaiah P, et al. Development and Preliminary Evaluation of a Multivariate Index Assay for Ovarian Cancer. PLoS ONE. 2009;4(2):e4599.
38. Macciò A, Madeddu C, Massa D, Astara G, Farci D, Melis GB, et al. Interleukin-6 and leptin as markers of energy metabolicchanges in advanced ovarian cancer patients. Journal of Cellular and Molecular Medicine. 2009;13(9b):3951-9.
39. Lutgendorf SK, Weinrib AZ, Penedo F, Russell D, DeGeest K, Costanzo ES, et al. Interleukin-6, cortisol, and depressive symptoms in ovarian cancer patients. J Clin Oncol. 2008;26(29):4820-7.

40. Kavsak PA, Lee A, Hirte H, Young E, Gauldie J. Cytokine elevations in acute coronary syndrome and ovarian cancer: a mechanism for the up-regulation of the acute phase proteins in these different disease etiologies. Clin Biochem. 2008;41(7-8):607-10.
41. Lambeck AJA, Crijns APG, Leffers N, Sluiter WJ, Ten Hoor KA, Braid M, et al. Serum Cytokine Profiling as a Diagnostic and Prognostic Tool in Ovarian Cancer: A Potential Role for Interleukin 7. Clinical Cancer Research. 2007;13(8):2385-91.
42. Chechlinska M, Kaminska J, Markowska J, Kramar A, Steffen J. Peritoneal fluid cytokines and the differential diagnosis of benign and malignant ovarian tumors and residual/recurrent disease examination. Int J Biol Markers. 2007;22(3):172-80.
43. Costanzo ES, Lutgendorf SK, Sood AK, Anderson B, Sorosky J, Lubaroff DM. Psychosocial factors and interleukin-6 among women with advanced ovarian cancer. Cancer. 2005;104(2):305-13.
44. Macciò A, Madeddu C, Massa D, Mudu MC, Lusso MR, Gramignano G, et al. Hemoglobin levels correlate with interleukin-6 levels in patients with advanced untreated epithelial ovarian cancer: role of inflammation in cancer-related anemia. Blood. 2005;106(1):362-7.
45. Gorelik E. Multiplexed Immunobead-Based Cytokine Profiling for Early Detection of Ovarian Cancer. Cancer Epidemiology Biomarkers & Prevention. 2005;14(4):981-7.
46. Daraï E, Detchev R, Hugol D, Quang NT. Serum and cyst fluid levels of interleukin (IL) -6, IL-8 and tumour necrosis factor-alpha in women with endometriomas and benign and malignant cystic ovarian tumours. Hum Reprod. 2003;18(8):1681-5.
47. Dobryszycka W, Katnik-Prastowska I, Gerber J, Lemańska K, Utko K, Rozdolski K. Serum haptoglobin, CA 125 and interleukin 6 levels in malignant and non-malignant tumors of the ovary. Arch Immunol Ther Exp (Warsz). 1999;47(4):229-36.
48. Tempfer C, Zeisler H, Sliutz G, Haeusler G, Hanzal E, Kainz C. Serum Evaluation of Interleukin 6 in Ovarian Cancer Patients. Gynecologic Oncology. 1997;66(1):27-30.
49. Scambia G, Testa U, Benedetti Panici P, Foti E, Martucci R, Gadducci A, et al. Prognostic significance of interleukin 6 serum levels in patients with ovarian cancer. Br J Cancer. 1995;71(2):354-6.
50. Plante M, Rubin SC, Wong GY, Federici MG, Finstad CL, Gastl GA. Interleukin-6 level in serum and ascites as a prognostic factor in patients with epithelial ovarian cancer. Cancer. 1994;73(7):1882-8.
51. Schröder W, Ruppert C, Bender HG. Concomitant measurements of interleukin-6 (IL-6) in serum and peritoneal fluid of patients with benign and malignant ovarian tumors. European journal of obstetrics, gynecology, and reproductive biology. 1994;56(1):43-6.
52. van der Zee AG, de Cuyper EM, Limburg PC, de Bruijn HW, Hollema H, Bijzet J, et al. Higher levels of interleukin-6 in cystic fluids from patients with malignant versus benign ovarian tumors correlate with decreased hemoglobin levels and increased platelet counts. Cancer. 1995;75(4):1004-9.
53. Moradi MM, Carson LF, Weinberg B, Haney AF, Twiggs LB, Ramakrishnan S. Serum and ascitic fluid levels of interleukin-1, interleukin-6, and tumor necrosis factor-alpha in patients with ovarian epithelial cancer. Cancer. 1993;72(8):2433-40.
54. Gastl G, Plante M, Finstad CL, Wong GY, Federici MG, Bander NH, et al. High IL-6 levels in ascitic fluid correlate with reactive thrombocytosis in patients with epithelial ovarian cancer. Br J Haematol. 1993;83(3):433-41.
55. Berek JS, Chung C, Kaldi K, Watson JM, Knox RM, Martínez-Maza O. Serum interleukin-6 levels correlate with disease status in patients with epithelial ovarian cancer. Am J Obstet Gynecol. 1991;164(4):1038-42; discussion 42-3.
